# O.6.1-7 Self-evaluation survey supporting student participation in physical activity promotion in lower secondary schools

**DOI:** 10.1093/eurpub/ckad133.266

**Published:** 2023-09-11

**Authors:** Katja Rajala, Harto Hakonen, Kati Lehtonen, Katariina Kämppi

**Affiliations:** Jamk University of Applied Sciences, Likes, Finland; katja.rajala@jamk.fi; Jamk University of Applied Sciences, Likes, Finland; katja.rajala@jamk.fi; Jamk University of Applied Sciences, Likes, Finland; katja.rajala@jamk.fi; Jamk University of Applied Sciences, Likes, Finland; katja.rajala@jamk.fi

## Abstract

**Purpose:**

A self-evaluation survey for students was developed in 2021 as a part of the national programme called Finnish Schools on the Move (FSM). The purpose of the survey is to increase and strengthen student participation in developing a physically active operating culture in schools. Using the survey, schools get information about adolescents’ physical activity (PA) during the school day as well as adolescents’ ideas and wishes concerning how to increase PA in the school environment.

**Methods:**

The online survey available on the FSM programme’s website has six statements related to adolescents’ participation in PA during the school day: break-time activities in the schoolyard and inside the school, break-time activities directed by other students or to other students, active commuting, and exercise breaks in lessons. The survey also contains 14 statements related to students’ opinions and experiences on operations increasing PA during the school day. In addition, the survey includes two open-ended questions through which students can describe what would motivate them to participate in PA and how they would increase their PA in school. The survey is available to all lower secondary schools and the schools receive a summary of students’ responses.

**Results:**

By the end of February 2023, the data consist of 2,506 self-evaluations from 11 schools. The results varied between schools and reflected the practices of the school’s operating culture. On the other hand, certain topics were repeated in the responses of all students, for example, related to factors that motivate them to engage in PA in school: peer support, better opportunities for PA around the schoolyard, rewards for PA, exercise breaks during lessons, interesting sport equipment, and access to the gym during break-times.

**Conclusions:**

To increase PA especially in lower secondary schools, students could be given more opportunities to take part in planning and implementing different kinds of activities. The self-evaluation survey for students is one way to gather students’ opinions and strengthen their participation in the development of actions increasing PA during the school day.

**Support/Funding Source:**

The Ministry of Education and Culture of Finland.

